# Dexmedetomidine Ameliorated Cognitive Dysfunction Induced by Intestinal Ischemia Reperfusion in Mice with Possible Relation to the Anti-inflammatory Effect Through the Locus Coeruleus Norepinephrine System

**DOI:** 10.1007/s11064-022-03706-w

**Published:** 2022-08-09

**Authors:** Gang Li, Jun Zhou, Jicheng Wei, Bin Liu

**Affiliations:** 1 Department of Anesthesiology, West China Hospital of Sichuan University, Chengdu, China; 2 Department of Anesthesiology, Affiliated Hospital of Southwest Medical University, Luzhou, China

**Keywords:** Dexmedetomidine, Cognitive dysfunction, Neuroinflammation, Locus coeruleus noradrenergic system, Intestinal ischemia/reperfusion injury

## Abstract

Cognitive impairment is a common central nervous system complication that occurs following surgery or organs damage outside the nervous system. Neuroinflammation plays a key role in the molecular mechanisms of cognitive impairment. Dexmedetomidine alleviates neuroinflammation and reduces cognitive dysfunction incidence; however, the mechanism by which dexmedetomidine alleviates cognitive dysfunction remains unclear. This study evaluated the effect of dexmedetomidine on attenuation of early cognitive impairment induced by intestinal ischemia–reperfusion in mice and examined whether the locus coeruleus norepinephrine (LCNE) system participates in the anti-inflammatory effect of dexmedetomidine. The superior mesenteric artery was clamped for 45 min to induce intestinal ischemia reperfusion injury. Dexmedetomidine alone or combined with DSP-4, a selective locus coeruleus noradrenergic neurotoxin, was used for pretreatment. Postoperative cognition was assessed using the Morris water maze. Serum and hippocampal levels of IL-1β, TNF-α, norepinephrine (NE), and malondialdehyde (MDA) were assessed by enzyme-linked immunosorbent assay. Immunofluorescence, immunohistochemistry, and hematoxylin and eosin staining were used to evaluate the expression of tyrosine hydroxylase (TH) in the locus coeruleus, hippocampal microglia, and intestinal injury. Pretreatment with dexmedetomidine alleviated intestinal injury and decreased the serum and hippocampal levels of NE, IL-1β, TNF-α, and MDA at 24 h after intestinal ischemia reperfusion, decreased TH-positive neurons in the locus coeruleus, and ameliorated cognitive impairment. Similarly, DSP-4 pre-treatment alleviated neuroinflammation and improved cognitive function. Furthermore, α2-adrenergic receptor antagonist atipamezole or yohimbine administration diminished the neuroprotective effects and improved cognitive function with dexmedetomidine. Therefore, dexmedetomidine attenuated early cognitive dysfunction induced by intestinal ischemia–reperfusion injury in mice, which may be related to its anti-inflammatory effects through the LCNE system.

## Introduction

Cognitive impairment is a common central nervous system complication that occurs following surgery or tissue and organ damage outside the nervous system. It is characterized by the progressive deterioration of intellectual/cognitive function and manifests as mental derangement, memory loss, anxiety, personality changes, and learning decline following anesthesia and surgery [1–3]. Cognitive dysfunction can increase mortality, decrease quality of life, prolong hospitalization, and increase the burden on the medical care system [4]. Studies have shown that neuroinflammation caused by surgery or tissues and organs damage outside the nervous system plays a key role in the molecular mechanism of cognitive dysfunction [5, 6]. Surgical trauma and intestinal ischemia reperfusion can lead to a systemic inflammatory response, resulting in the release of damage-associated molecular patterns, followed by leukocyte recruitment and release of inflammatory cytokines. Overexpressed cytokines, such as tumor necrosis factor (TNF)-α, cross the blood–brain barrier, increase neuroinflammation, interfere with neuronal activity, and eventually lead to cognitive decline. Therefore, regulation of neuroinflammation can treat cognitive dysfunction [7–9].

Dexmedetomidine is a highly selective α2-adrenoceptor agonist with sedative and anti-anxiety effects and is widely used in clinical anesthesia and intensive care unit departments [10]. Previous studies have shown that dexmedetomidine has a protective effect on multiple organs, including the lungs, heart, and kidney [11–13]. In some animal models of systemic inflammatory response syndrome and surgical trauma, dexmedetomidine lessened the severity of neuroinflammation and neuronal apoptosis [14–17], therefore reducing the incidence of cognitive dysfunction [17, 18]. However, the mechanism by which dexmedetomidine alleviates cognitive dysfunction remains unclear. The locus coeruleus (LC) is the main noradrenergic nucleus in the brain and is a crucial regulatory site of nociceptive nerve transmission.

The neuronal terminals of the LC project to almost the entire central nervous system (CNS), releasing norepinephrine (NE) in several functional brain regions, including the hippocampus [19, 20]. NE released from LC neurons regulates a wide range of advanced cognitive functions by binding to receptors. Norepinephrine fibers in the cortex and hippocampus can participate in the regulation of learning, memory, psychiatric disorders, and emotion [21]. Degeneration or damage of the LC leads to a decrease in the level of NE in the projection area. Chronic neuroinflammation can decrease tyrosine hydroxylase (TH) expression in LC neurons and norepinephrine levels in projection areas such as the cortex and hippocampus, leading to cognitive impairment and Alzheimer’s disease [22]. Recent studies have shown that the locus coeruleus norepinephrine (LCNE) system plays a critical role in central inflammatory injury. Norepinephrine (NE) released from LC neurons modulates different aspects of neuroinflammation. Therefore, LC may be a novel target for regulating inflammation [23]. The locus coeruleus is a target of dexmedetomidine in the brain [24, 25]. Some studies have confirmed that dexmedetomidine can decrease norepinephrine release and cell firing in locus coeruleus slices of rats, and that it attenuates ischemia-induced increases in striatal norepinephrine concentrations [26, 27]. Therefore, we hypothesized that the anti-inflammatory effect of dexmedetomidine in the CNS is mediated by the LCNE system.

Previous research has demonstrated that intestinal ischemia–reperfusion (I/R) injury can induce inflammatory changes in the brain and impair cognitive function [28–30]. The present study was designed to (1) evaluate the effect of cognition induced by intestinal I/R injury in mice; (2) investigate the changes in the levels of inflammation factors IL-1β or TNF-α and the levels of norepinephrine or MDA in the hippocampus and peripheral serum; and (3) explore the alteration of microglia density in the CNS when dexmedetomidine or DSP-4 (N-(2-chloroethyl)-N-ethyl-2-bromobenzylamine hydrochloride, a selective locus coeruleus-noradrenergic neurotoxin, was administered. DSP-4 can easily pass through the blood–brain barrier and accumulate into noradrenergic nerve terminals via the norepinephrine transporters. At the dose of 50 mg/kg intraperitoneal injection, it is characterized by a rapid and long-lasting loss of noradrenaline but not in the peripheral sympathetic nerve and non-locus coeruleus norepinephrine system. Because of the selectivity for the locus coeruleus system, DSP-4 is often used as a useful tool in the study of the functional role of noradrenergic system in the brain [31–33].

## Materials and Methods

### Experiment 1

#### Animals and Experimental Groups

Animals used in this study were exclusively C57BL/6J mice. Healthy male mice (aged 8–10 weeks, weighing 20–25 g,) were maintained under a normal 12-h light–dark cycle with ad libitum access to food and water. All animals were allowed to adapt to their environment for 7–10 days before the experiment. All animals were fasted for 12 h prior to the experiments but had free access to water. Mice were randomly assigned to the following six groups (n = 12 in each group): (a) control (CON), (b) sham (SHAM), (c) intestinal I/R, (d) dexmedetomidine pretreatment before intestinal I/R (DEX + I/R), (e) DSP-4 pre-treatment before intestinal I/R (DSP-4 + I/R), and (f) DSP-4 and dexmedetomidine pretreatment before intestinal I/R (DSP-4 + DEX I/R). Mice in the CON group received phosphate-buffered saline (PBS) to control for the effects of injection stress. The intestinal I/R group underwent intestinal ischemia for 45 min under general anesthesia and then reperfusion for 24 h [34]. In brief, mice were anesthetized with pentobarbital (40 mg/kg intraperitoneally injected, IP), the small intestine was exteriorized by a 1-cm midline abdominal incision, and the superior mesenteric artery (SMA) was clamped with a microvascular clip. Ischemic injury was initiated, and the wound was covered with sterile cotton gauze. After 45 min of ischemia, the SMA was reperfused. Bupivacaine (0.25%) was infiltrated, and the wound was closed using sterile sutures. SHAM animals underwent the same procedure as the intestinal I/R group, but without SMA clamping. In the DEX + I/R group, mice were administered dexmedetomidine hydrochloride (Yangzijiang Medical Corporation, China) 50 μg/kg IP 30 min before intestinal ischemia. The dosage of dexmedetomidine was chosen based on a previous study [35]. On days 7 and 14 before intestinal I/R injury, mice in the DSP-4 + I/R group were administered DSP-4 (N-(2-chloroethyl)-N-ethyl-2-bromobenzylamine hydrochloride, #C8417, Sigma-Aldrich Corporation, St Louis, MO, USA) at 50 mg/kg IP. The dose and timepoint of DSP-4 have been used in many previous studies [36, 37]. Animals in the DSP-4 + DEX + I/R group received DSP-4 and then dexmedetomidine before intestinal I/R, and the dose and time points were the same as those in the DEX + I/R or DSP-4 + I/R groups. All procedures were performed in accordance with the National Institutes of Health guidelines for the use of experimental animals. Ethical approval number: swmu20210428.

After 24 h of reperfusion, mice were sacrificed after spatial working (Morris water maze [MWM]) tests. Hippocampal tissues and serum of half of the animals (n = 6) in each group were quickly harvested and stored at − 80 °C until they were used for subsequent ELISA testing. The remaining animals (n = 6) in each group were sacrificed after deep isoflurane anesthesia and perfused transcardially with ice-cold saline followed by 4% paraformaldehyde solution in PBS (pH 7.4). The intestine was stained with hematoxylin and eosin (HE) for morphological examination using Chiu’s score method [38]. The brains were dissected and post-fixed in 4% paraformaldehyde in PBS overnight and then dehydrated by floating on 15% sucrose and then 30% sucrose successively, followed by immunofluorescence or immunohistochemistry staining.

#### Morris Water Maze

To evaluate hippocampus-dependent memory, the MWM test was used as previously reported [39, 40]. During the training session, mice were first put into the water from one of the four quadrants and trained to find the hidden platform for 5 consecutive days. If the platform was not located during 60 s, mice were guided to the platform and allowed to stay there for 30 s. The time taken by mice to find the hidden platform (escape latency) was recorded. Animals underwent intestinal I/R surgery on day 6. After 24 h, the probe test was applied to assess spatial memory. The submerged platform was removed, and the number of platform crossings, time spent in the target quadrant, and average swimming speed were recorded. A video camera mounted on the ceiling, directly above the center of the maze, was used in conjunction with an animal tracking system (XINRUAN Information Technologies, Shanghai, China).

#### Immunofluorescence

Fixed and dehydrated intact mouse brains were placed on a horizontal surface and vertically blocked into the anterior and posterior regions along the posterior margin of the bilateral cerebral cortices. The posterior part of the mouse brains was embedded in OCT compound, then 35-μm thick brain slices containing LC nuclei were collected. One out of every four consecutive sections in the locus coeruleus area was taken for TH (TH, rabbit, 1:400, #PAB9649, Abnova Corporation, Taipei, Taiwan) immunofluorescent staining according to the method of Cao [41]. The collected brain slices were rinsed in Tris-buffered saline. The donkey anti-rabbit IgG antibody conjugated with Alexa Fluor 594 (1:200, Invitrogen, Waltham, MA, USA) was chosen as the secondary antibody. Sections were incubated with secondary antibody for 2 h at room temperature in the dark. Then, an Olympus IX73 fluorescence microscope (Olympus Corp., Tokyo, Japan) was used to observe and take photographs with a 590-nm wavelength. TH- positive cells in the locus coeruleus were counted with Image Pro Plus 6.0 software(Media Cybernetics, Rockville, ML, USA). ​The mean of TH- positive cells in slices per mouse was statistically analyzed [42].

#### Immunohistochemistry

The anterior part of the brain was embedded in paraffin and sectioned for immunohistochemical analysis. After deparaffinization and antigen retrieval, 5-μm thick coronal sections containing the cerebral cortex and hippocampus were incubated with 3% H_2_O_2_ in methanol at room temperature for 10 min to block endogenous peroxidase. After sections were washed in PBS, background was blocked in PBS containing 1% bovine serum albumin at room temperature for 1 h. Subsequently, the brain sections were incubated with ionized calcium binding adapter molecule 1 (Iba-1) antibody (rabbit, 1:800, #17198S, Cell Signaling Technology, Danvers, MA, USA) at 4 °C overnight and then incubated in the presence of secondary antibody (1:500, Abcam, Cambridge, UK). Three sections were imaged per mouse (Olympus IX73). Image Pro Plus 6.0 software was used to count the Iba-1 positive cells/mm2 in the CA1 region of the hippocampus, and the mean of three sections was taken for statistical analysis.

#### Norepinephrine, Inflammatory Cytokines, or Malondialdehyde Determination in the Hippocampus or Serum by ELISA

Mice were euthanized by deep isoflurane anesthesia at 24 h after intestinal I/R surgery. Blood samples were collected from the right ventricle after thoracotomy. After being placed for 20 min at room temperature, blood was centrifuged at 3000×*g* for 20 min at 4 °C, and serum was collected. After blood collection, bilateral hippocampi were harvested after perfusion with cold saline. The hippocampi were dissected on ice. The levels of NE and inflammatory cytokines IL-1β, TNF-α, and malondialdehyde (MDA) in the hippocampi and serum were determined using commercial ELISA kits (Meimian Technology Corporation, China). This method was performed according to the manufacturer’s instructions.

The serum inflammatory cytokine and NE levels are expressed as pg/mL and ng/mL, respectively. The levels of NE and inflammatory cytokines in the hippocampus were normalized to the total protein content and are presented as pg/mg or ng/mg protein. MDA is presented as nmol/mL in the serum or nmol/mg protein in the hippocampus.

## Experiment 2

To further evaluate whether the anti-inflammatory effect of dexmedetomidine in the CNS is mediated by α2-adrenergic receptors, 60 C57BL/6J mice were randomly divided into five groups: SHAM, IR, dexmedetomidine (DEX), and two α2-adrenergic antagonists, atipamezole (ATI) and yohimbine (YOH). The method and dose were the same as those used for the SHAM, IR, and DEX groups. The other two groups received atipamezole hydrochloride at 500 μg/kg (#B7226, APExBIO, Taipei, Taiwan) or yohimbine hydrochloride at 2 mg/kg (#N1704, APExBIO,) intraperitoneally at 30 min before dexmedetomidine injection. The levels of NE, IL-1β, TNF-α, and MDA in the hippocampus were determined by ELISA. The MWM was used to test cognitive function. MDA is expressed as nmol/mg of protein in the hippocampus.

### Statistical Analysis

All data were analyzed using SPSS version 25.0 (IBM Corp., Armonk, NY, USA). Levene's test was used to test the homogeneity of the variances among the groups. As all data were normally distributed, statistical data are expressed as the means ± standard errors of the mean. Data generated from repeated measures of latencies in the training section of the MWM were analyzed using two-way repeated measures ANOVA. Other data were analyzed using one-way ANOVA, followed by Tukey’s post-hoc multiple comparison tests. Significance was established a priori as *p* < 0.05.

## Results

## Experiment 1

### Dexmedetomidine Ameliorated Intestinal Mucosal Injury Following Ischemia/Reperfusion and Improves Chiu’s Score

**Fig. 1:**
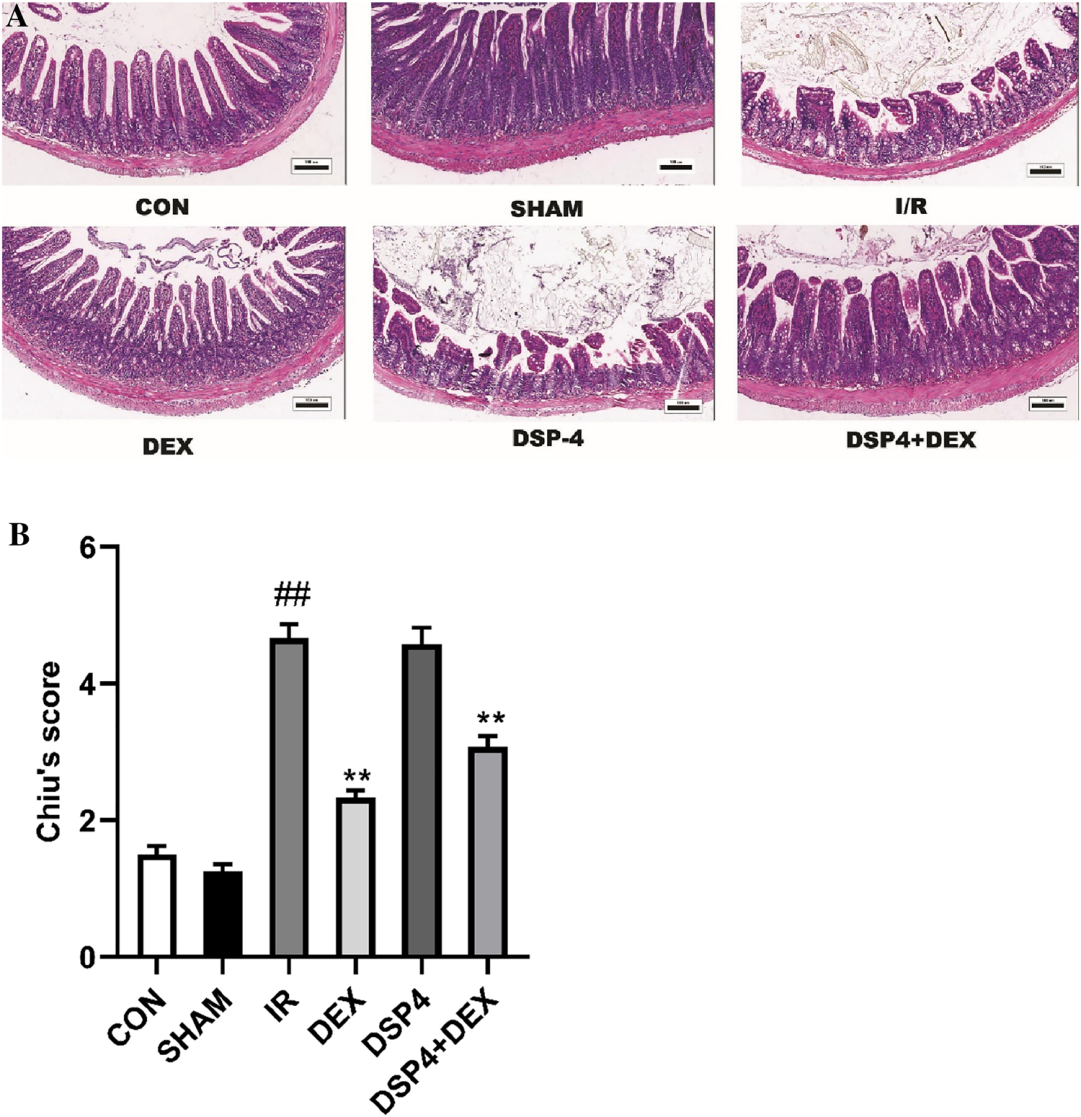
Dexmedetomidine ameliorates the intestinal mucosal injury following ischemia/reperfusion (I/R). **A** Histopathological changes of the intestinal mucosa. Representative sections were taken in the CON and SHAM groups 24 h after intestinal ischemia/reperfusion (I/R) with different interventions (hematoxylin and eosin staining × 100, scale bars = 100 μm). **B** Evaluation of intestinal injury under light microscopy using Chiu’s scores 24 h after intestinal I/R with different interventions. *CON* control group, *SHAM* sham group, *I/R* intestinal I/R group, *DEX* dexmedetomidine pretreatment then intestinal I/R group, *DSP-4* DSP-4 pretreatment then intestinal I/R group, *DSP-4* + *DEX* DSP-4 and dexmedetomidine pretreatment then intestinal I/R group. Data are expressed as mean ± standard error of the mean (n = 6). ##*p* < 0.01 versus SHAM group, **p < 0.01 versus I/R group

To confirm the protective effect of dexmedetomidine on the intestinal mucosa after intestinal I/R, the intestine was stained with HE for morphological examination using Chiu’s score method. As shown in Fig. 1A, B, the intestines of mice in the CON and SHAM groups exhibited normal mucosal architecture. However, of the six groups, Chiu’s scores were found to have significant differences by ANOVA (F (5, 30) = 79.81, p < 0.01). Compared with the SHAM group, intestinal I/R caused marked intestinal damage and a higher Chiu’s score in the I/R group (*p* < 0.01). Compared with the I/R group, DSP-4 pretreatment did not alleviate ischemia/reperfusion injury of the intestine (*p* = 0.9992); however, dexmedetomidine pretreatment significantly ameliorated intestinal mucosal injury and decreased Chiu’s score in the DEX (*p* < 0.01) and DSP-4 + DEX groups (*p* < 0.01).

### Dexmedetomidine Decreased the Number of Tyrosine Hydroxylase-Positive Cells in the Locus Coeruleus and Lowered Norepinephrine Level in the Hippocampus and Serum After Intestinal Ischemia/reperfusion

**Fig. 2:**
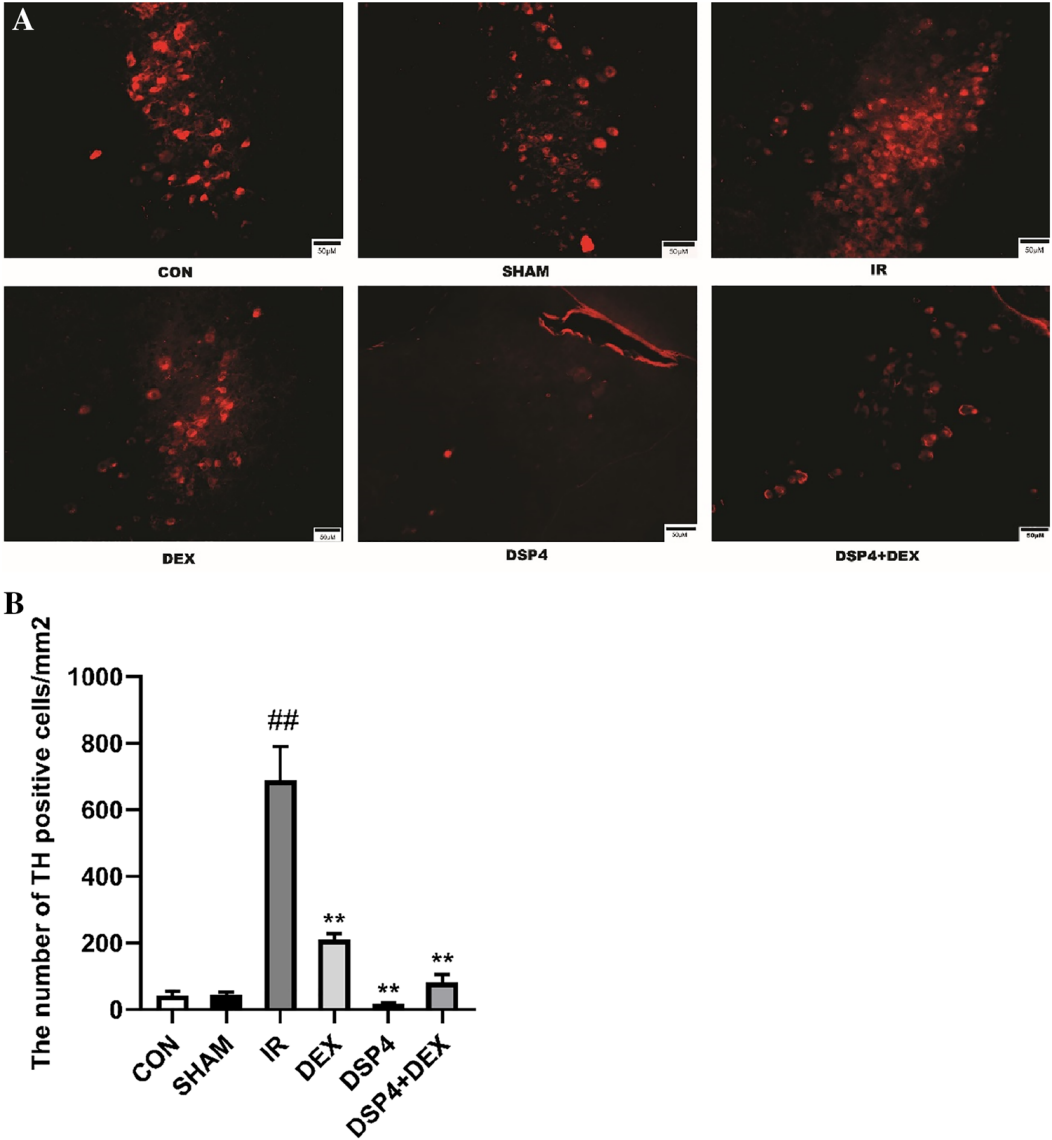
Dexmedetomidine decreases the number of tyrosine hydroxylase-positive cells in the locus coeruleus. **A** Tyrosine hydroxylase (TH)-positive cells in the locus coeruleus (immunofluorescent staining × 200, scale bars = 50 μm). **B** Number of TH-positive cells in the locus coeruleus. *CON* control group, *SHAM* sham group, *IR* intestinal ischemia/reperfusion (I/R) group, *DEX* dexmedetomidine (DEX) + I/R group, *DSP-4*, DSP-4 + I/R group, *DSP-4* + *DEX* DSP-4 + DEX + I/R group. Data are expressed as mean ± standard error of the mean (n = 6). ##*p* < 0.01 versus SHAM group, **p < 0.01 versus IR group

**Fig. 3:**
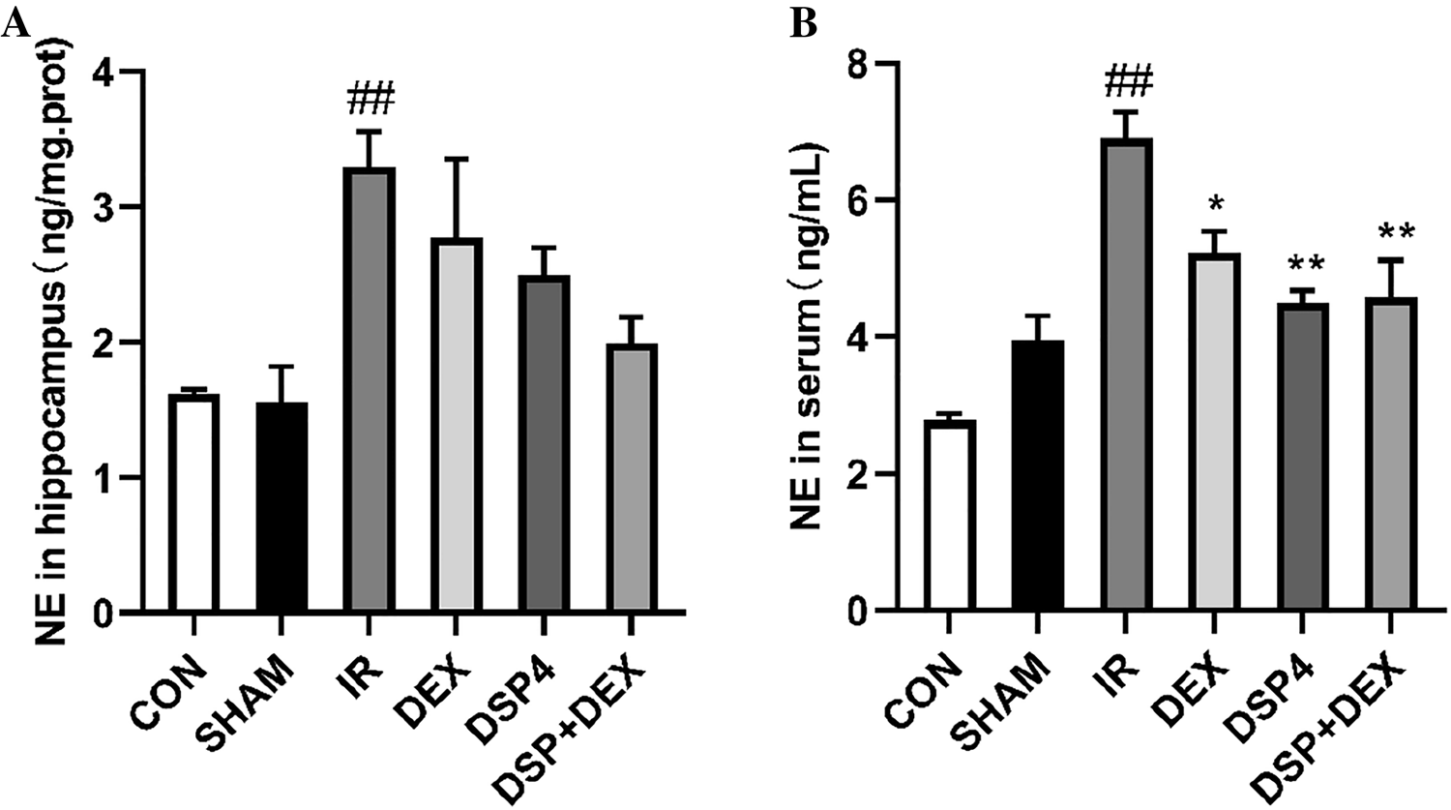
Norepinephrine (NE) levels in the hippocampus (**A**) and serum (**B**). NE level was measured by ELISA. *CON* control group, *SHAM* sham group, *IR* intestinal ischemia/reperfusion (I/R) group, *DEX* dexmedetomidine (DEX) + I/R group, *DSP-4* DSP-4 + I/R group, *DSP-4* + *DEX* DSP-4 + DEX + I/R group. Data are expressed as mean ± standard error of the mean (n = 6). ##*p* < 0.01 versus SHAM group, *p < 0.05 versus IR group, **p < 0.01 versus IR group

As shown in Figs. 2A, B and 3A, B, at 24 h after intestinal I/R injury, the TH-positive cells in the LC were found to have significant differences among the groups, as calculated by One Way ANOVA (overall F (5, 30) = 36.11, *p* < 0.01). Compared with the CON or SHAM groups, TH-positive cells in the LC of the I/R group significantly increased (*p* < 0.01). However, compared with the I/R group, the TH-positive cells of the LC in the DEX, DSP-4, and DSP-4 + DEX groups significantly decreased (*p* < 0.01). Between all six groups, NE was also found to have significant differences by One Way ANOVA in the hippocampus(overall (F (5, 30) = 4.20, *p* < 0.01) or serum (overall (F (5, 30) = 11.67, *p* < 0.01). Compared with the SHAM groups, NE of the I/R group significantly increased in the hippocampus or serum (both *p* < 0.01). Compared with the I/R group, serum NE levels significantly decreased in the DEX group (*p* < 0.05) and in the DSP-4 and DSP-4 + DEX groups (both *p* < 0.01).

### Dexmedetomidine Lowered Inflammatory Cytokine and Malondialdehyde Levels in the Hippocampus and Serum

**Fig. 4:**
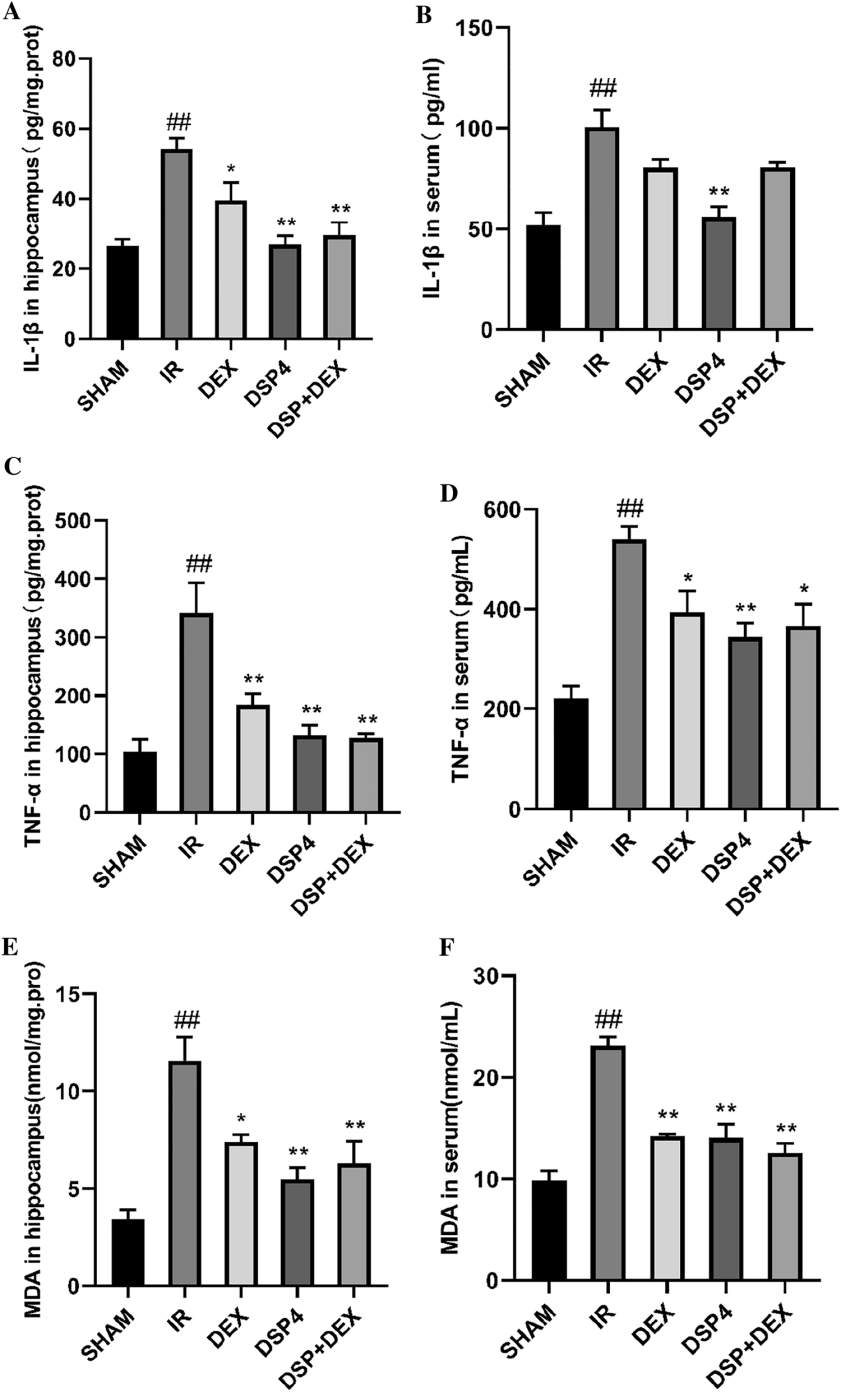
Inflammatory cytokines and malondialdehyde levels in the hippocampus and serum. IL-1β levels in the hippocampus (**A**) and serum (**B**); TNF-α levels in the hippocampus (**C**) and serum (**D**); MDA levels in the hippocampus (**E**) and serum (**F**). IL-1β, TNF-α, and malonaldehyde levels were measured by ELISA. *SHAM* sham group, *IR* intestinal ischemia/reperfusion (I/R) group, *DEX* dexmedetomidine (DEX) + I/R group, *DSP-4* DSP-4 + I/R group, *DSP-4* + *DEX* DSP-4 + DEX + I/R group. Data are expressed as mean ± standard error of the mean (n = 6). ##*p* < 0.01 versus SHAM group, *p < 0.05 versus IR group, **p < 0.01 versus IR group

As shown in Fig. 4A–F, at 24 h following intestinal I/R injury, between all five groups, IL-1β, TNF-α, and MDA levels were found to have significant differences by One Way ANOVA (IL-1β: hippocampus [overall F (4, 25) = 11.61, *p* < 0.01], serum [overall F (4, 25) = 12.87, *p* < 0.01]; TNF-α: hippocampus [overall F (4, 25) = 11.86, *p* < 0.01], serum [overall F (4, 25) = 11.40, *p* < 0.01]; MDA: hippocampus [overall F (4, 25) = 12.99, *p* < 0.01], serum [overall F (4, 25) = 28.80, *p* < 0.01]). Compared with the SHAM group, IL-1β, TNF-α, and MDA levels significantly increased in the I/R group in the hippocampus (*p* < 0.01) or serum (*p* < 0.01).Compared with the I/R group, IL-1β, TNF-α, and MDA levels in the DEX group significantly decreased (IL-1β: hippocampus(*p* < 0.05); TNF-α: hippocampus (*p* < 0.01), serum(*p* < 0.05); MDA: hippocampus(*p* < 0.05), serum *p* < 0.01). Interestingly, compared with the I/R group, IL-1β, TNF-α, and MDA levels significantly decreased in the DSP-4 and DSP-4 + DEX groups (IL-1β: hippocampus in DSP-4 or DSP-4 + DEX (both *p* < 0.01), serum in DSP-4 ( *p* < 0.01); TNF-α: hippocampus in DSP-4 or DSP-4 + DEX(both *p* < 0.01), serum in DSP-4 (*p* < 0.01) or DSP-4 + DEX( *p* < 0.05); MDA: hippocampus in DSP-4 or DSP-4 + DEX (both *p* < 0.01), serum in DSP-4 or DSP-4 + DEX(both *p* < 0.01).

### Dexmedetomidine Ameliorated Intestinal Ischemia/Reperfusion-Induced Memory Impairment in Mice

**Fig. 5:**
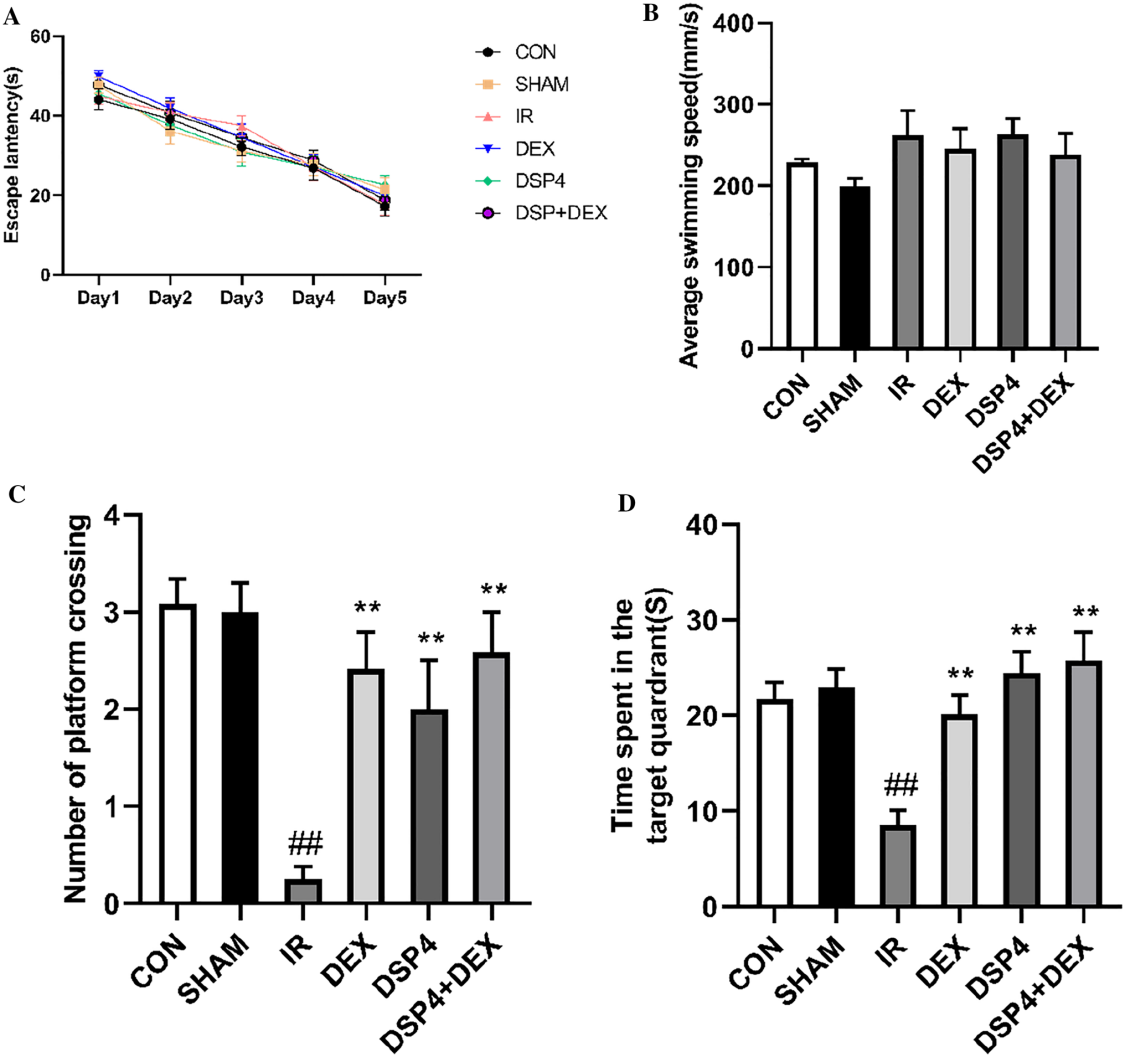
Dexmedetomidine ameliorates intestinal ischemia/reperfusion injury-induced learning and memory impairment in mice. **A** Average escape latency for the training session in the Morris water maze (MWM) test. **B** Swimming speed 24 h following intestinal ischemia/reperfusion (I/R) injury. **C** Number of platform-crossing in the probe testing of MWM with hidden platform removed. **D** Time spent in the target quadrant in the probe testing of MWM with hidden platform removed. Data are presented as mean ± standard error of the mean. ##*p* < 0.01 versus the SHAM group, **p < 0.01 versus the intestinal ischemia/reperfusion injury group. n = 12 in each group

As shown in Fig. 5A–D, the protective effect of dexmedetomidine on memory impairment induced by intestinal I/R was explored using the MWM. In the training session of the MWM test, no significant difference was observed in the escape latency among the groups (Fig. 5A, overall F (5, 66) = 0.683, *p* = 0.637). This indicated that all experimental mice were able to search for the hidden platform after training. During the MWM probe session, the average swimming speed was not significantly different between the groups (Fig. 5B, F (5, 66) = 1.215, *p* > 0.05). Between all six groups, the number of platform crossings was found to have a significant difference by One Way ANOVA (Fig. 5C, overall F (5, 66) = 8.726, *p* < 0.01), and the time spent in the target quadrant further had a significant difference (Fig. 5D, overall F (5, 66) = 8.562, *p* < 0.01). Compared with the CON or SHAM group, the number of platform crossings and the time spent in the target quadrant were remarkably reduced in the IR group (*p* < 0.01), suggesting that intestinal I/R injury could cause memory impairment. Nevertheless, memory impairment induced by intestinal I/R was ameliorated by dexmedetomidine treatment (Fig. 5C, D, p < 0.05). Simultaneously, we found that memory impairment induced by intestinal I/R was significantly ameliorated by preconditioning with DSP-4 or DSP-4 + DEX (Fig. 5C, D, p < 0.01).

### Dexmedetomidine Prevented the Increase in Microglial Density Induced by Intestinal Ischemia/Reperfusion

**Fig. 6:**
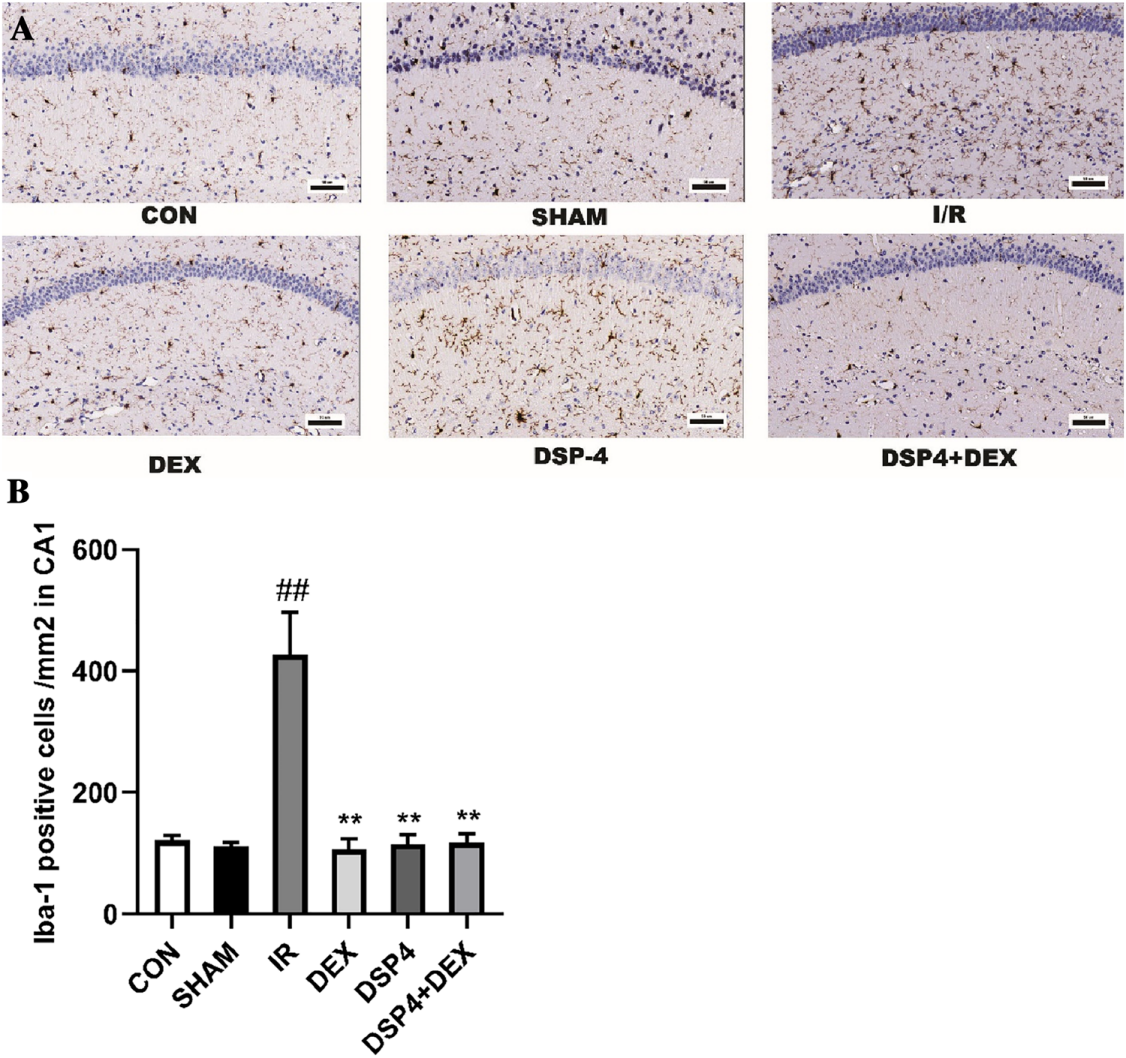
Dexmedetomidine suppresses intestinal I/R injury-induced microglial activation. **A** Representative sections of the CA1 region of the hippocampus were obtained from the control (CON) and sham (SHAM) groups 24 h after intestinal ischemia/reperfusion injury with different interventions (immunohistochemistry staining × 200, scale bars = 50 μm). **B** Quantification of Iba-1-positive microglia in the CA1 region. Data are expressed as mean ± standard error of the mean (n = 6). ##*p* < 0.01 versus SHAM group, **p < 0.01 versus IR group

Microglial activation plays a key role in neuroinflammation. Immunohistochemistry was performed, and Iba-1-positive cells in the CA1 region of the hippocampus were counted. As shown in Fig. 6A, B, the density of Iba-1-positive cells in the CA1 region was found to have significant differences by One Way ANOVA (overall F (5, 30) = 16.88, *p* < 0.01). Compared with the CON or SHAM groups, the density of Iba-1-positive cells in the I/R group significantly increased (*p* < 0.01). However, compared with the I/R group, the density of Iba-1-positive cells in the DEX, DSP-4 and DSP-4 + DEX groups significantly decreased (*p* < 0.01).

## Experiment 2

### Dexmedetomidine Lowered Inflammatory Cytokines or Malonaldehyde in the Hippocampus, and the Effects were Diminished by an α2-Adrenergic Antagonist

**Fig. 7:**
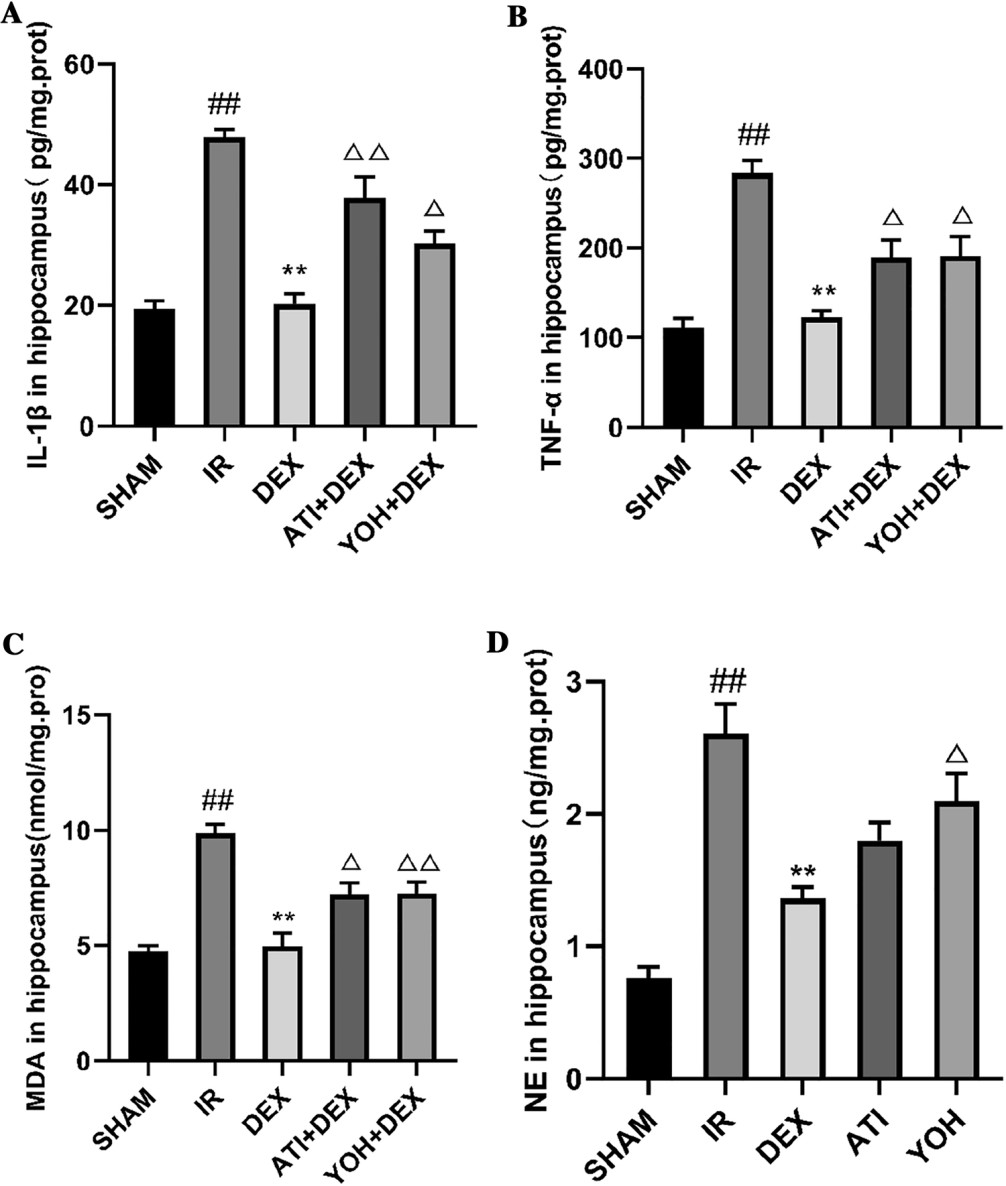
IL-1β, TNF-α, malondialdehyde and NE levels in the hippocampus. IL-1β (**A**), TNF-α (**B**), malondialdehyde (**C**) and NE levels (**D**) were measured using ELISA. *SHAM* sham group, *IR* intestinal ischemia/reperfusion I/R group, *DEX* dexmedetomidine (DEX) + I/R group, *ATI* + *DEX* atipamezole and dexmedetomidine pretreatment followed by intestinal I/R, *YOH* + *DEX* yohimbine and dexmedetomidine pretreatment followed by intestinal I/R group. Data are expressed as mean ± standard error of the mean (n = 6). ##*p* < 0.01, versus SHAM group; **P < 0.01, versus IR group; △*P* < 0.05, versus DEX group; △△*P* < 0.01, versus DEX group

As shown in Fig. 7A–D, similar to the results of Experiment 1, after 24 h of intestinal I/R injury, among the five groups, IL-1β, TNF-α, MDA, and NE levels in the hippocampus were found to have significant differences on one-way ANOVA (IL-1β: [overall F (4, 25) = 31.95, *p* < 0.01]; TNF-α: [overall F (4, 25) = 19.37, *p* < 0.01]; MDA: [overall F (4, 25) = 22.00, *p* < 0.01] and NE: [overall F (4, 25) = 19.45, *p* < 0.01]). Compared with the SHAM group, IL-1β, TNF-α, MDA and NE levels significantly increased in the hippocampus of the I/R group (*p* < 0.01). Compared to the I/R group, IL-1β, TNF-α, MDA, and NE levels in the DEX group significantly decreased in the hippocampus (*p* < 0.01). However, compared with the DEX group, IL-1β, TNF-α, MDA and NE levels in the hippocampus significantly increased in the ATI + DEX (IL-1β, *p* < 0.01; TNF-α, *p* < 0.05; MDA, *p* < 0.05) or YOH + DEX groups (IL-1β, *p* < 0.05; TNF-α, *p* < 0.05; MDA, *p* < 0.01; NE, *p* < 0.05).

### Dexmedetomidine Ameliorated Intestinal Ischemia/Reperfusion-Induced Memory Impairment in Mice, and the Effects were Diminished by an α2-Adrenergic Antagonist

**Fig. 8:**
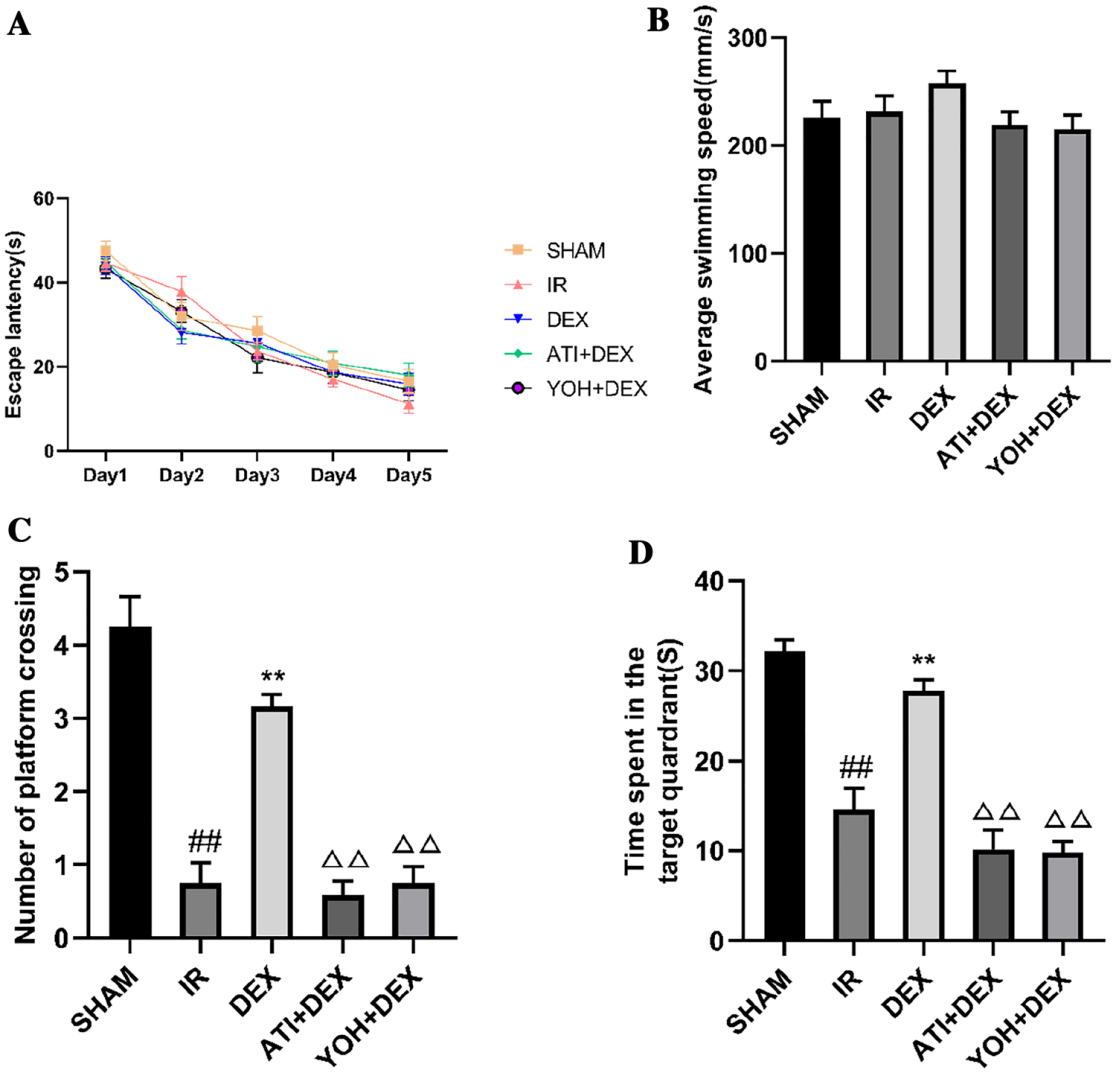
Dexmedetomidine ameliorates intestinal ischemia/reperfusion injury-induced learning and memory impairment in mice. **A** Average escape latency for the training session in the Morris water maze (MWM) test. **B** Swimming speed 24 h following intestinal ischemia/reperfusion (I/R) injury. **C** Number of platform crossings in the probe testing of MWM with hidden platform removed. **D** Time spent in the target quadrant in the probe testing of MWM with hidden platform removed. Data are presented as mean ± standard error of the mean. ##*p* < 0.01 versus the SHAM group, **p < 0.01 versus the IR group, △△*P* < 0.01 versus DEX group. n = 12 in each group

As shown in Fig. 8A–D, the protective effect of dexmedetomidine on intestinal I/R-induced memory impairment was explored using the MWM. In the training session of the MWM test, no significant differences were observed in the escape latency between the groups (Fig. 8A, overall F (4, 55) = 0.588, *p* = 0.672). During the MWM probe session, the average swimming speed was not significantly different between the groups (Fig. 8B, F (4, 55) = 1.572, *p* > 0.05). Of the five groups, the number of platform crossings was found to have a significant difference by One Way ANOVA (Fig. 8C, overall F (4, 55) = 40.11, *p* < 0.01), and the time spent in the target quadrant had a significant difference (Fig. 8D, overall F (4, 55) = 35.84, *p* < 0.01). Compared with the SHAM group, the number of platform crossings and the time spent in the target quadrant were remarkably reduced in the IR group (*p* < 0.01); however, the intestinal I/R-induced memory impairment was ameliorated by dexmedetomidine treatment (Fig. 8C, D, *p* < 0.01). Moreover, compared with the DEX group, memory impairment significantly deteriorated following preconditioning with atipamezole (Fig. 8C, D, *p* < 0.01) or yohimbine (Fig. 8C, D, *p* < 0.01).

## Discussion

This study aimed to investigate the effects of dexmedetomidine on neuroinflammation and cognitive impairment induced by intestinal I/R injury in mice. We found that IL-1 β and TNF-α levels in the hippocampus and peripheral serum increased at 24 h following intestinal ischemia/reperfusion injury; additionally, the number of crossings of the target platform and the time spent in the target quadrant both decreased in mice in the I/R group that underwent the MWM test, thus confirming that intestinal I/R injury could aggravate CNS inflammation and lead to cognitive dysfunction. These results confirm those of previous studies[27, 43, 44]. Dexmedetomidine could reduce the incidence of cognitive impairment after intestinal I/R injury, which may be related to decreased levels of inflammatory cytokines IL-1β, IL-6, C-reactive protein, and TNF-α [45]. Our study found that at 24 h after the intestinal I/R injury, preconditioning with dexmedetomidine significantly reduced intestinal mucosal injury and improved Chiu's score in mice with intestinal I/R injury. Compared to intestinal I/R mice, IL-1β, TNF-α, and MDA levels in the hippocampus and peripheral serum of mice pretreated with dexmedetomidine significantly decreased. The results of the MWM suggested that cognitive function was improved, which confirmed that dexmedetomidine could reduce intestinal inflammation and neuroinflammation caused by intestinal I/R, improve cognitive dysfunction, and protect damaged organs. This may be related to the anti-inflammatory and antioxidative effects of dexmedetomidine. This result was consistent with those of previous research[46, 47].

Accumulating research findings show that the LCNE system is involved in neuroinflammation. Nonetheless, the role of LCNE in regulation of inflammation remains controversial[23, 48, 49]. To evaluate whether dexmedetomidine improved neuroinflammation and cognitive function through the LCNE system after intestinal ischemia/reperfusion injury in mice, we used the neurotoxic drug DSP-4 to selectively degrade LC norepinephrine neurons at 2 weeks and 1 week before operation, which was confirmed by immunofluorescence staining showing a significant decrease in TH-positive neurons in the LC. We found that 24 h following intestinal I/R, both the TH expression in the LC and the NE level in the hippocampus or serum increased in the I/R group, whereas the TH expression and the level of NE in the hippocampus or serum decreased in the dexmedetomidine preconditioning group. After administration of DSP-4, the NE level in the serum decreased; the IL-1 β, TNF-α, and MDA levels in the hippocampus decreased; the cell density of microglia in the hippocampus decreased; and the number of crossing target platforms and the time spent in the target quadrant increased in the MWM test. These findings suggest that DSP-4 can cause degeneration of NE terminals and neurons in the locus coeruleus, thus decreasing the density of microglia in the CNS, alleviating inflammation, and improving hippocampal-dependent memory. These results confirmed that damaging the function of LC in the brain had an important anti-inflammatory effect on intestinal I/R injury in mice and improved cognitive function. Interestingly, we found that NE of the hippocampus in the DSP-4 group was not significantly different from that in the IR group. We postulated that the reasons may be as follows: first, some studies have proven that when the DSP-4 was used, NE of the relevant brain regions decreased by 60–80%. This may be attributed to the fact that the residual TH-positive neurons release NE to the hippocampus during intestinal ischemia/reperfusion injury [49, 50]. However, it remains unclear whether there is a release of NE from the brain region of the non-locus coeruleus to the hippocampus during intestinal ischemia/reperfusion [51]. This should be examined in future research.

These findings were consistent with those reported by Wang et al., who showed that DSP-4 suppressed neuroinflammation caused by intestinal ischemia and attenuated cognitive impairment [49]. When dexmedetomidine was administered after DSP-4 administration, we found that DSP-4 could not attenuate the protective effect of dexmedetomidine on hippocampal inflammatory injury, and dexmedetomidine did not further enhance or weaken the effects of DSP-4. We believe that this is because both DSP-4 and dexmedetomidine can inhibit the NE neurons in the LC and reduce the amount of NE in the brain. As a specific inhibitor of LC noradrenergic neurons, DSP-4 is more effective in reducing NE and inflammation than the current dose of dexmedetomidine; therefore, it does not demonstrate further anti-inflammatory and cognitive improvement effects. Therefore, we used the α2-adrenergic receptor blockers atipamezole and yohimbine to observe the effects of dexmedetomidine on the anti-inflammatory and cognitive functions of the CNS. The results showed that the anti-inflammatory and cognitive improvement effects of dexmedetomidine significantly diminished in the presence of α2-adrenergic receptor blockers. Compared with the dexmedetomidine group, IL-1β, TNF-α, and MDA levels in the hippocampus of the atipamezole and yohimbine groups increased, and cognitive impairment was aggravated. In summary, our results suggest that dexmedetomidine preconditioning ameliorates neuroinflammation and cognitive impairment induced by intestinal I/R in mice. This mechanism may be related to the activation of α2-adrenergic receptor in the CNS, inhibition of the LCNE system, and reduction of norepinephrine in the brain, thus relieving neuroinflammation.

The adrenergic and cholinergic systems are intertwined. The cholinergic system is activated when the adrenergic system is inhibited. Studies have shown that vagus nerve stimulation attenuates the systemic inflammatory response and that dexmedetomidine prevents renal I/R injury, depending in part on cholinergic anti-inflammatory mechanisms [52, 53]. In our study, TH-positive neurons in the LC pretreated with dexmedetomidine were inhibited, and norepinephrine as an adrenergic neurotransmitter was also reduced in the CNS. This may stimulate the cholinergic anti-inflammatory pathway in the CNS, resulting in anti-inflammatory effects and improved cognitive function. Therefore, dexmedetomidine attenuates early cognitive dysfunction induced by intestinal ischemia/reperfusion injury in mice, which may be related to its anti-inflammatory effect through the LCNE system. Accordingly, the LCNE system may be a potential target for regulating neuroinflammation and preventing cognitive impairment, which requires further study.

## Data Availability

Data are available upon reasonable request.
